# DNA metabarcoding highlights cyanobacteria as the main source of primary production in a pelagic food web model

**DOI:** 10.1126/sciadv.adg1096

**Published:** 2023-04-26

**Authors:** Andreas Novotny, Baptiste Serandour, Susanne Kortsch, Benoit Gauzens, Kinlan M. G. Jan, Monika Winder

**Affiliations:** ^1^ Department of Ecology, Environment and Plant Sciences, Stockholm University, Stockholm, Sweden.; ^2^ Spatial Foodweb Ecology Group, Department of Agricultural Sciences, University of Helsinki, Helsinki, Finland.; ^3^ Environmental and Marine Biology, Åbo Akademi University, Turku 20500, Finland.; ^4^ German Centre for Integrative Biodiversity Research (iDiv) Halle-Jena-Leipzig, Leipzig, Germany.; ^5^ Institute of Biodiversity, Friedrich Schiller University, Jena, Germany.

## Abstract

Models that estimate rates of energy flow in complex food webs often fail to account for species-specific prey selectivity of diverse consumer guilds. While DNA metabarcoding is increasingly used for dietary studies, methodological biases have limited its application for food web modeling. Here, we used data from dietary metabarcoding studies of zooplankton to calculate prey selectivity indices and assess energy fluxes in a pelagic resource-consumer network. We show that food web dynamics are influenced by prey selectivity and temporal match-mismatch in growth cycles and that cyanobacteria are the main source of primary production in the investigated coastal pelagic food web. The latter challenges the common assumption that cyanobacteria are not supporting food web productivity, a result that is increasingly relevant as global warming promotes cyanobacteria dominance. While this study provides a method for how DNA metabarcoding can be used to quantify energy fluxes in a marine food web, the approach presented here can easily be extended to other ecosystems.

## Teaser

A flux-based model incorporating molecular diet data reveals the importance of feeding selectivity in shaping food web dynamics.

## INTRODUCTION

Energetic food web models are used by decision-makers globally to manage marine populations, predict the effects of environmental change on commercial fish stocks, and inform control of algal blooms (*1*). While marine food web productivity are determined by diverse and dynamic interactions of prey (phytoplankton) and predators (zooplankton) at the base of the food webs (*2*, *3*), most food web models are poorly resolved at lower trophic levels (*4*). With shifting plankton communities, there is a pressing need to account for species-specific feeding interactions, but a lack of empirical knowledge of these interactions is a major limiting factor for constructing resolved food webs, particularly between primary producers and primary consumers (*5*–*7*). Consequently, it remains unknown to what extent different primary producers are contributing to food web productivity at higher trophic levels and how food web structure and ecosystem functioning will respond to changes in primary production (*8*).

Global warming alters pelagic food webs by favoring cyanobacteria over eukaryotic phytoplankton (*9*, *10*). In eutrophic waters, the abundance of large-sized filamentous cyanobacteria (around 20 to 1000 μm) has been increasing, affecting water quality (*5*–*7*). In addition, the increasing success of small-sized picocyanobacteria (<2 μm) in open waters is assumed to cause declines in trophic efficiency (*9*, *11*) and food web degradation (*12*). Although being major primary producers in the oceans, many food web models assume that cyanobacteria are not fed upon by zooplankton due to their size and sometimes toxicity (*13*, *14*). On the contrary, recent evidence from tracing studies suggests that cyanobacteria are incorporated in food webs (*15*–*20*), but it remains unclear to what extent. Without a resolved food web model accounting for the diverse feeding selectivity of zooplankton, the functional role and consequence of increasing cyanobacteria in marine ecosystems will remain unclear.

Obtaining the detailed information on feeding selectivity of zooplankton (or predator-prey preferences) needed to quantify energy fluxes in highly resolved food webs and thereby assess the role of cyanobacteria would require running selectivity laboratory experiments for all pairwise interactions in a food web (*21*–*23*). This is something that quickly becomes unfeasible in complex communities that vary over space and time. DNA metabarcoding is increasingly used to detect trophic interactions in various ecosystems (*24*, *25*) and has become an important tool for identifying potential predator-prey interactions in network models (*26*, *27*). However, as the output of metabarcoding is not directly proportional to prey biomass (*24*, *28*, *29*), DNA metabarcoding has until now not been used to calculate predator-prey preferences and energy fluxes in complex food webs.

**Fig. 1.**
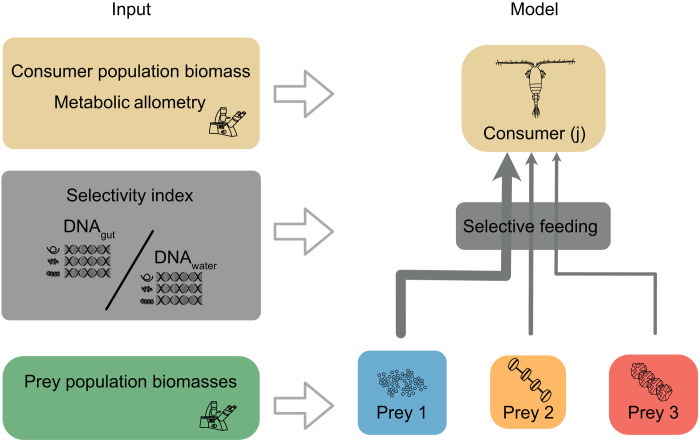
Combining DNA metabarcoding, species population biomasses, and metabolic allometry to estimate energy fluxes in a bioenergetic model. We used a bioenergetic model to calculate energy fluxes between zooplankton (consumers) and phytoplankton (resources) in a pelagic food web of the Baltic Sea. The model builds on a steady-state assumption, where total energy gains for each consumer equals their energetic losses. Energy is claimed from resources depending on their availability (e.g., biomasses), assimilation efficiencies, and consumer preferences or selectivity for each resource. Population biomasses and body mass information of the consumers were retrieved from the Swedish national pelagic monitoring database. Resource selectivity indices for each consumer were calculated using DNA metabarcoding of zooplankton gut content and water samples. This information was used to weigh the energy fluxes in the food web model. For a more detailed description, see Materials and Methods.

In this study, we used diet information from DNA metabarcoding to calculate the relative feeding selectivity of zooplankton (*30*) and thus overcome the limitations of previous studies to quantify fluxes of energy in a bioenergetic food web model (*31*) (Fig. 1). We used the 16*S rRNA* gene metabarcoding of zooplankton consumers collected during several seasons at three different stations in the Baltic Sea proper (fig. S1). This approach provides a model with high trophic resolution and enables us to identify the main sources and pathways of primary production in a pelagic food web. The DNA analyses and the model revealed that cyanobacteria are incorporated into pelagic food webs, a result that urges us to reconsider the structural and functional role of cyanobacteria in present-day and future marine food webs.

## RESULTS

**Fig. 2.**
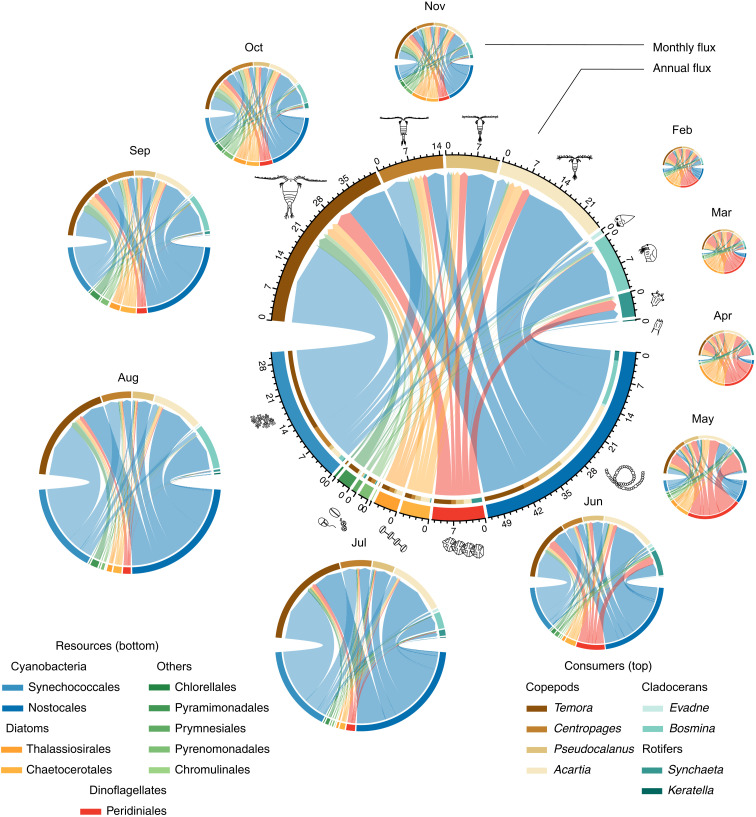
Consumer-resource network of the pelagic Baltic Sea. Link width is proportional to fluxes of energy (kJ/m^2^) between resources (phytoplankton, bottom) and consumers (zooplankton, top). The width of the nodes (taxa) corresponds to each population’s contribution to annual secondary production. The diameter of each plot is proportional to the square root of the total production.

Our consumer-resource network for the Baltic Sea proper included eight of the most abundant zooplankton genera of several taxonomic groups and size classes, including copepods (*Temora*, *Centropages*, *Pseudocalanus*, and *Acartia*), cladocerans (*Evadne* and *Bosmina*), and rotifers (*Synchaeta* and *Keratella*) (Fig. 2). We included 10 orders of primary producers identified as resource items for the zooplankton by *16*S *rRNA* gene (*16*S) metabarcoding and being abundant in the Baltic Sea biomass monitoring. The phytoplankton orders included diatoms (Chaetocerales and Thalassiosirales) and dinoflagellates (Peridiniales) that together constitute the spring bloom; filamentous cyanobacteria (Nostocales) and picocyanobacteria (Synechococcales) that together constitute the summer bloom; as well as chlorophytes (Chlorellales and Pyramimonadales), haptophytes (Prymnesiales), cryptophytes (Pyrenomonadales), and chrysophytes (Chromulinales) (Fig. 2).

Our bioenergetic model, used to calculate fluxes of energy between phytoplankton and zooplankton for all days of the year, revealed a dynamic food web structure, where both links and total food web productivity varied over the seasons (Fig. 2 and table S1). The total detected annual energy flux from primary producers to primary consumers was 114 kJ/m^2^ per year. Among this flux, filamentous cyanobacteria (Nostocales, 46%) and picocyanobacteria (Synechococcales, 26%) together constituted the major source of primary production in the food web. Spring bloom-forming dinoflagellates (Peridiniales, 10%) and diatoms (Chaetocerotales and Thalassiosirales, 11%), as well as a diversity of other small-sized phytoplankton (6%), contributed to a lesser extent to the detected annual secondary production (Fig. 2).

In our model, the copepod *Temora* was the consumer responsible for the largest proportion of the annual secondary production (37%). *Temora* accounted for most of the total detected predation on Synechococcales (65%) and other groups of small phytoplankton (82% of Pyraminomonadales, 62% of Chromulinales, and 53% of Chlorellales). At the same time, *Temora* accounted for a relatively small proportion of the detected predation on filamentous cyanobacteria (19% of Nostocales) and chain-forming diatoms (25% of Chaetocerotales). The copepods *Centropages* and *Pseudocalanus* accounted for 13 and 10% of the detected secondary production, respectively, and appeared to split their prey equally between available resources (Fig. 2), while the copepod *Acartia* and the cladoceran *Bosmina* accounted for 23 and 11% of the detected secondary production and had the strongest grazing impact on filamentous cyanobacteria (33 and 21% of the total grazing on Nostocales, respectively). Although cladoceran and rotifer zooplankton (*Evadne*, *Bosmina*, *Synchaeta*, and *Keratella*) had comparatively low-energy consumption due to relatively low biomass throughout the year, they together constituted 17% of the detected annual predation on primary producers (Fig. 2).

**Fig. 3.**
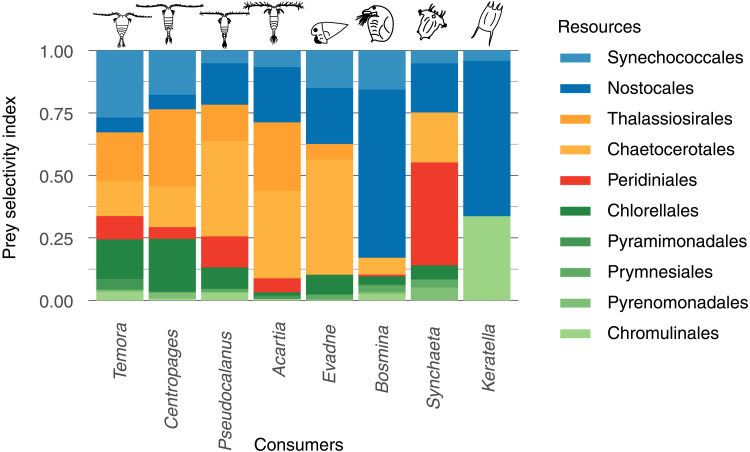
Predator-prey selectivity index for the consumers (zooplankton) in the food web calculated from *16*S *rRNA* gene read abundance.

**Fig. 4.**
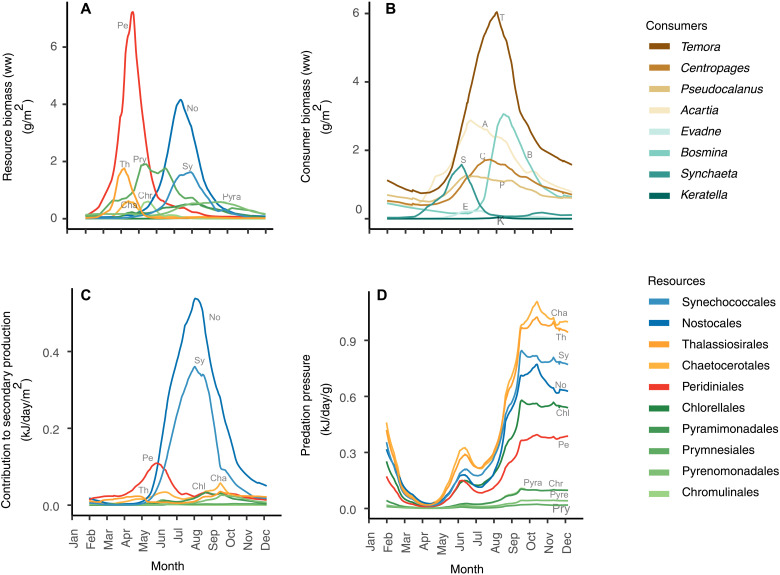
Seasonal dynamics in the Baltic Sea pelagic food web. Daily population biomasses (g/m^2^) of (**A**) resources (phytoplankton) and (**B**) consumers (zooplankton). (**C**) Contribution of each resource to daily food web secondary production (kJ/m^2^). (**D**) Daily predation pressure for each resource population (kJ/g).

The annual fluxes of energy in the food web were driven both by zooplankton-specific prey selectivity (Fig. 3; also when accounting for uncertainty, see fig. S2) and by strong fluctuations in the biomass of resources (Fig. 4A) and consumers (Fig. 4B). Selectivity indices calculated from *16*S metabarcoding revealed a clear differentiation in forage niche between different zooplankton genera (Fig. 3). For instance, several of the non-copepod zooplankton (such as *Keratella* and *Bosmina*) had a higher preference for filamentous cyanobacteria (Nostocales) compared to the copepods *Temora* and *Centropages*. Similarly, copepods had a generally higher preference for diatoms than the non-copepod zooplankton (Fig. 3). A temporal mismatch between the spring bloom of phytoplankton in March–May and the major peak in zooplankton biomass in summer (June–August) resulted in a relatively low contribution to secondary production by the dinoflagellate order Peridiniales (Fig. 4B) that had the highest biomass among the phytoplankton in spring. Peridiniales was the main resource for the rotifer *Synchaeta*, which peaked in biomass during the spring bloom (Figs. 2B and 4B). In contrast to the low biomasses of zooplankton observed during the spring bloom, the summer bloom of cyanobacteria co-occurred with the peak in zooplankton biomass. The mismatch is also reflected in the predation pressure on phytoplankton that was lowest during the spring months when biomasses of phytoplankton were high and increased toward the end of the summer bloom when zooplankton had their highest abundance (Fig. 4D).

## DISCUSSION

The outcome of this study shows that prey preferences of different zooplankton genera have big effects on the consumption of primary production within the food web, stressing the importance of assessing and integrating predator-prey preferences in food web models. Our study also shows that filamentous cyanobacteria and picocyanobacteria are the two major sources of primary production for upper trophic levels, a consequence of both prey preferences and temporal predator-prey co-occurrence. This knowledge demonstrates that cyanobacteria can make a substantial contribution to secondary production beyond that coming from diatoms and dinoflagellates.

Studies that estimate energy fluxes typically assume that zooplankton groups cannot graze efficiently on cyanobacteria (*12*, *32*, *33*) due to their body size (*34*). However, when using in situ DNA observations of zooplankton feeding selectivity, our model shows that some major groups of zooplankton indeed graze efficiently on the cyanobacteria summer bloom. These findings are supported by both experimental studies, showing that zooplankton can shorten cyanobacteria filaments (*35*, *36*) and more recent observations, on the basis of DNA and tracing approaches, indicating that cyanobacterial matter is incorporated in the food webs (*15*–*19*). While these studies were all able to detect trophic transfer, they did not quantify the relative contribution of cyanobacterial production in contrast to other important phytoplankton sources. Our model shows that the phytoplankton spring bloom only contributes marginally to ecosystem productivity and that the Baltic Sea food web mainly is based on cyanobacteria primary production. This is supported by a study quantifying input to the seafloor, concluding that fresh organic matter originating from the phytoplankton spring bloom constitutes the major input of the sediment, followed by more degraded matter (fecal pellets and zooplankton body parts) during the summer bloom (*37*).

Our approach provides a way forward for integrating DNA sequencing in food web models. A previous limiting factor in pelagic food web models aiming at calculating energy fluxes was to assess the weights of trophic interactions at the base of the food web (*23*). By using DNA metabarcoding to calculate feeding selectivity of consumers, we were able to account for the diverse trophic niches of several zooplankton genera. While selectivity calculations rely on the relative difference between gut content and prey availability, it is not affected by quantitative biases between DNA sequences and prey biomass that until now have limited the use of metabarcoding for energy flux quantification (*24*, *28*, *29*). Hence, our study bridges the gap between DNA metabarcoding and energetic food web modeling. Still, models taking variation in prey selectivity, different taxonomic levels, rare species, and life stages into account will be important directions for quantifying the seasonal and spatial energy fluxes from primary producers to consumers. However, as DNA sequencing is increasingly used in monitoring, our approach has the potential to be applied to many different ecosystems.

Understanding how different sources of primary production are channeled through the food web is becoming increasingly important as global warming is altering the composition of primary producers. As observed in many systems, blooms of cyanobacteria have increased, which is currently suggested to cause declines in biological productivity (*12*, *38*) and rapid and widespread expansion of hypoxic bottoms (*39*–*41*). Considering the feeding preferences of zooplankton, our study shows that a large fraction of this cyanobacterial primary production is being efficiently channeled to the zooplankton community. This result calls for a revision in our understanding of cyanobacteria’s role in food webs and suggests a reevaluation of current marine management plans for the spread of hypoxia and predictions of future ocean productivity.

## MATERIALS AND METHODS

### Zooplankton sampling and DNA metabarcoding

Zooplankton was sampled at three locations in the Baltic Sea (Landsort Deep, Gotland Deep, and Bornholm Deep) over the season between 2017 and 2020 (fig. S1). Zooplankton samples were collected with vertical hauls from 0 to 30 m and 30 to 60 m using a 90-μm WP2 plankton net and preserved immediately in 95% ethanol. Individuals of zooplankton were identified and isolated under a stereomicroscope (table S2). To remove contamination of external DNA, all individuals were rinsed five times in Milli-Q water, screened for visible epibionts, soaked for 30 s in a 1% bleach solution, and rinsed another five times in Milli-Q water. Five to 12 individuals of the same species were pooled randomly into the same sample tube and stored with 180 μl of alanine transaminase lysis buffer (QIAGEN, Hilden, Germany). For estimating available prey composition, water samples were collected with 10-liter Niskin bottles with 5-m depth intervals above the thermocline of 30-m depth or with a 20-m long hose according to the Helsinki Commission’s guidelines for plankton monitoring in the Baltic Sea (*42*). The depths were mixed by adding an equal volume of water from the Niskin bottles. A total of one to three liters were sequentially filtered onto 25-mm-diameter filters with 20-μm, 2-μm (polycarbonate), and 0.2-μm (nylon) pore sizes.

DNA from pooled zooplankton individuals was extracted using the QIAamp DNA Micro Kit (QIAGEN), including 1 μg of carrier RNA following the manufacturer’s instructions for tissue samples. DNA from water samples was extracted from the water filters using the DNeasy Plant Mini Kit (QIAGEN) with an additional step of bead beating with 1-mm glass beads and an overnight incubation at 56°C with proteinase K (QIAGEN). Illumina sequencing library preparation was performed according to best practices (*43*). For each step in the library preparation procedure (DNA extraction to amplification), a negative control was included. After the full library preparation, the negative controls were analyzed with Qubit and gel electrophoresis and did not result in observable bands.

We amplified a 500–base pair (bp)–long fragment of the V3-V4 region of the *16*S *rRNA* gene (*16*S) using the primers 341F-Adapter1 and 805R-Adapter2 (table S3) (*44*, *45*). The polymerase chain reaction (PCR) reactions contained 10 μl of HiFi HotStart Ready Mix (Roche, KAPA Biosystems, Basel, Switzerland), 1 μl of each primer (10 nM; with attached adapter sequence) (Eurofins Genomics, Ebersberg, Germany), 2 μl of template DNA, and 6 μl of PCR grade water (QIAGEN). Thermal cycling conditions included 98°C of initial denaturation for 2 min, followed by 25 cycles of 98°C denaturation for 20 s, 63°C annealing for 20 s, 72°C elongation for 15 s, and a final extension step of 2 min at 72°C. PCR products were cleaned using Agencourt AMPure XP magnetic beads according to the manufacturer’s instructions (Beckman Coulter, Brea, CA).

An outer PCR step was added to attach unique index sequences, to facilitate sample pooling. Reactions contained 14 μl of KAPA HiFi HotStart Ready Mix (Roche, KAPA Biosystems), 1 μl of Handle1-index-Adapter1 (10 μM), 1 μl of Handle2-index-Adapter2 (10 μM), and 12 μl of cleaned PCR product from the previous step (table S3). The thermocycling conditions were 98°C for 2 min, followed by 10 cycles of 98°C for 20 s, 62°C for 30 s, 72°C for 30 s, and a final extension step of 2 min at 72°C. PCR products were pooled at equimolar amounts and purified using Agencourt AMPure XP (Beckman Coulter). DNA concentration and quality were determined using a Qubit fluorometer (Qubit dsDNA BR Assay, Thermo Fisher Scientific, Waltham, MA) and a Bioanalyzer assay (Agilent, Santa Clara, CA). Libraries were sequenced on MiSeq (MSC 2.5.0.5/RTA 1.18.54) pair-end setup (2 × 300 bp, version 3, Illumina, San Diego, CA) with the addition of 10% genomic PhiX.

MiSeq sequences were converted from Bcl to FastQ (Sanger/phred33/Illumina quality scale) using “bcl2fastq2” from the Casava software. Primers and adapters were truncated in the Cutadapt software, which also removes sequences where primers are missing *(**46**)*. All downstream analyses were conducted in R *(**47**).* Quality control and filtering, error rate modeling, sequence dereplication, ribosomal sequence variant inference, and taxonomic assignment were done using the DADA2 R package (*48*). Reads were truncated after 256 nucleotides (forward) and 220 nucleotides (reverse), and the two first nucleotides were removed. Sequences with maximum expected errors (EE) higher than 2 or sequences containing ambiguities (N) were removed. Pair ends were merged with a minimum overlap of 15 nucleotides, allowing for a maximum of one nucleotide mismatch. Sequences were taxonomically assigned using the Naive Bayesian Classifier (*49*) from the DADA2 R package to a custom-made database combining the SILVA *16*S reference database (*50*) with the PhytoREF database (*51*).

### Bias assumptions of selectivity index based on DNA metabarcoding

To assess the selectivity index (*S_i_*) for each prey taxon (*i*) of a consumer, we used a standardized forage ratio (*30*), which requires a quantitative estimate (e.g., biomass) of both the prey composition in the gut sample (*Bg_i_)* and the prey availability in the environment (*Be_i_*). The selectivity index for each prey can be calculated using the following equation (Eq. 1)

$$S_{i}=\frac{\frac{Bg_{i}}{Be_{i}}}{\sum_{i}\frac{Bg_{i}}{Be_{i}}}$$ (1)

Given that the same DNA metabarcoding protocol is used for assessing both prey composition of the gut and prey availability in the environment, we assume the following relationship between prey sequence read abundances (*Rg_i_* for gut samples and *Re_i_* for the environment) and prey biomass (*Bg_i_* for gut samples and *Be_i_* for the environment) (Eq. 2)

$$\left\{ \begin{array}{c} {Bg}_{i}=Rg_{i}*P_{i}*Lg\\ {Be}_{i}=Re_{i}*P_{i}*Le \end{array}\right.$$ (2)

Here, *P_i_* is a factor representing biases linked to the identity of the prey taxa (*i*), such as the ratio between tissue biomass and gene copy number (*25*); DNA recovery determining how much of the ingested DNA matter that can be recovered after sampling and DNA extraction (*52*); amplification efficiency (*53*); and other biases that remain constant independently of prey taxa. Similarly, *Lg* and *Le* represent biases that can be linked to the identity of the sample DNA library, including biases originating from differences in sample sizes, extraction efficiency (*54*), PCR performance (*55*), various steps of sample dilution, and others. Given that prey-related biases (*P*) are constant for both gut and environmental samples and that library-related biases (*L*) are independent of prey taxa, the selectivity index is not influenced by the values of either *P* or *L* (Eq. 3)

$$S_{i}=\frac{\frac{Rg_{i}*P_{i}*Lg}{Re_{i}*P_{i}*Le}}{\sum_{i}\frac{Rg_{i}*P_{i}*Lg}{Re_{i}*P_{i}*Le}}=\frac{\frac{Rg_{i}}{Re_{i}}}{\sum_{k}\frac{Rg_{i}}{Re_{i}}}$$ (3)

We used Eq. 3 to calculate selectivity indices for each zooplankton sample and prey taxon at sequence identity level. Selectivity indices for all zooplankton samples were summarized to class level. For all zooplankton species and prey classes, we calculated the mean of the selectivity indices (table S2).

### Biomasses and metabolic losses

The population biomasses of zooplankton and phytoplankton were retrieved from the Swedish Metrological and Hydrological Institute (SMHI: available at https://sharkweb.smhi.se) with sampling intensity ranging between monthly and weekly samples. Individual body masses of phytoplankton and zooplankton were retrieved from the Helsinki Commission’s guidelines for plankton monitoring in the Baltic Sea (*42*). We calculated daily biomass estimates over 1 year by linearly interpolating data from samples taken between 2007 and 2018.

The metabolic rate for each node was calculated on the basis of metabolic scaling theory (Eq. 4), where the metabolic rate *X_i_* (*J*/*s*) is derived from species’ body masses *M_i_* and an allometric scaling constant, i.e., a normalization constant *a* = 17.17 for invertebrates. *Be_i_* represents the total population biomass per area unit (grams per square meter) and is multiplied by the metabolic rate (flux per gram biomass) to obtain a metabolic rate estimate at the population level. Metabolic rates are also adjusted to ambient temperature, where *E* is the activation energy, *K* is Boltzmann’s constant, and *T* is the absolute temperature in Kelvin.

$$X_{i}=e^{\left(a*\ln M_{i}\right)}\times e^{\left(\frac{-E}{kT}+X_{0}\right)}\times {Be}_{i}$$ (4)

### Estimating energy fluxes

We used a bioenergetic model (*31*) to calculate energy fluxes (Joules per day per square meter) between all nodes (taxa) in the food web for each day of the year and each of the three stations. The model builds on a steady-state assumption (*56*, *57*), where the energy consumption of each species equals the energetic losses (*G_i_* = *L_i_*). In this model, losses of each population (*Li*) were defined as the sum of metabolic losses (*X_i_*) for the population and the energy flux lost to predation (*F_i_*) (Eq. 5). Gains for each population (*G_i_*) were calculated as the sum of all fluxes from prey to predator multiplied with the assimilation efficiency, i.e., conversion of consumed biomass into energy (*e_j_*) (Eq. 6) and an absolute prey preference constant *W_ij_*. The efficiency was based on the functional group of the prey and put to 0.77 (*58*). The prey preference was based on the selectivity index calculated from DNA abundances scaled with the relative biomass abundance of the prey species (Eq. 7). The fluxes were calculated using the “fluxing” function in the fluxweb R package version 0.2 (*31*)

$$L_{i}=X_{i}+F_{i}$$ (5)

$$G_{i}=\sum_{j}e_{ji}W_{ji}F_{ji}$$ (6)

$$W_{ij}=\frac{S_{ij}*{Be}_{i}}{\sum_{k}S_{kj}*{Be}_{k}}$$ (7)

$$\sum_{j}e_{ji}W_{ji}F_{ji}=X_{i}+\sum_{j}W_{ij}F_{ij}$$ (8)

### Influence of variation in biomass and prey selectivity

To account for annual variations in biomass of prey and predators, we estimated fluxes (Joules per day per meter squared) between primary producers and primary consumers for each day of the year and each station (*8*) by adjusting the biomass parameters for predators and prey. For each node and day of the year, we calculated the total predation losses as *F_i_* = ∑*_j_F_ij_* (Joules per day per meter squared), normalized predation pressure as *P_i_* = *F_i_*/*Be_i_* (Joules per gram per day) (*59*), and total consumption as *F_j_* = ∑*_i_F_ji_* (Joules per day per meter squared). We also calculated annual food web metrics by summarizing all daily flux networks (Joules per year per meter squared).

To test for the consistency of our results in regard to the variability in consumer preferences, we ran a bootstrapping analysis. For each consumer taxa *i*, our main results on selectivity were based on the average of *n_i_* replicates (table S2). One iteration of the bootstrap procedure consisted of random sampling with replacement of *n_i_* replicates for each consumer taxa *i* (using “slice_sample” from dplyr R package). We used this dataset to recalculate average preferences and corresponding energy fluxes. The procedure was repeated 100 times to characterize a distribution of fluxes showing the uncertainty around our predictions. Last, we compared the distributions against a null model that assumes no preferences (for each consumer the differences in foraging intensity depend only on its prey’s relative biomasses).

### Model limitations

Our model assumes a steady-state ecosystem where the energy required by zooplankton equals their resting state respiration. This assumption leads to an underestimation of secondary consumption when the population grows and overestimates the requirements during population decline. Furthermore, limited access to dietary data from DNA metabarcoding limited us to only include the most abundant taxa in the model. Including less abundant taxa and more trophic levels and life stages would influence the model output. Because of a limited resolution in the current taxonomic database used to annotate the *16*S sequences (*51*), the model was calculated on the order level. The development of more highly resolved taxonomic databases in the future will yield more robust measurements of selectivity. Last, while we account for systematic biases in DNA read abundance when calculating selectivity index (Eq. 3), nonsystematic biases may influence the result. For instance, primer competition biases that are dependent on the composition of prey taxa, rather than just species-specific amplification efficiency (*25*), were not accounted for in our approach. Likewise and similar to any observational study of gut contents, degradation rates that are unique both to the sample type and prey taxa were not accounted for in this model.
