# Supplementary material for: DNA metabarcoding highlights cyanobacteria as the main source of primary production in a pelagic food web model
Source: Sci Adv. 2023 Apr 26;9(17):eadg1096. doi: 10.1126/sciadv.adg1096 (PMC10132751; doi:10.1126/sciadv.adg1096)
Supplement: Supplementary file 1 — Figs. S1 and S2 Tables S1 to S3 Data S1 [file sciadv.adg1096_sm.pdf]

Science Advances
AAAS

Supplementary Materials for

DNA metabarcoding highlights cyanobacteria as the main source of primary production in a pelagic food web model

Andreas Novotny *et al.*

Corresponding author: Andreas Novotny, mail@andreasnovotny.se; Monika Winder, monika.winder@su.se

Sci. Adv. **9**, eadg1096 (2023)
DOI: 10.1126/sciadv.adg1096

This PDF file includes:

Figs. S1 and S2
Tables S1 to S3
Data S1

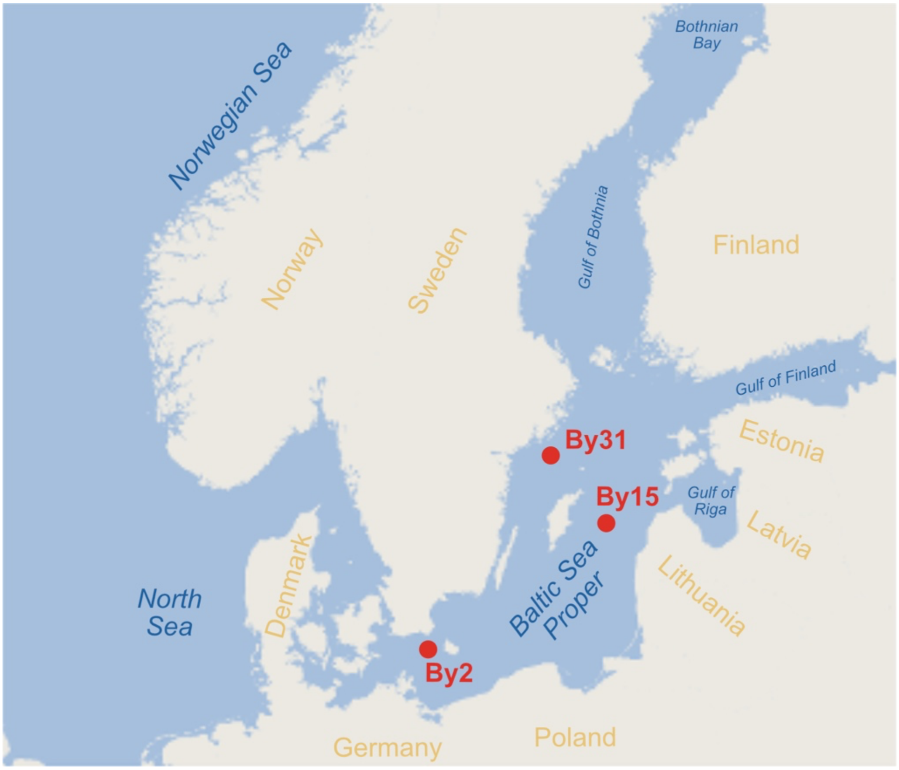

Fig. S1: Map of sampling stations. The map is retrieved from the Swedish national archive for oceanographic data: https://sharkweb.smhi.se/.

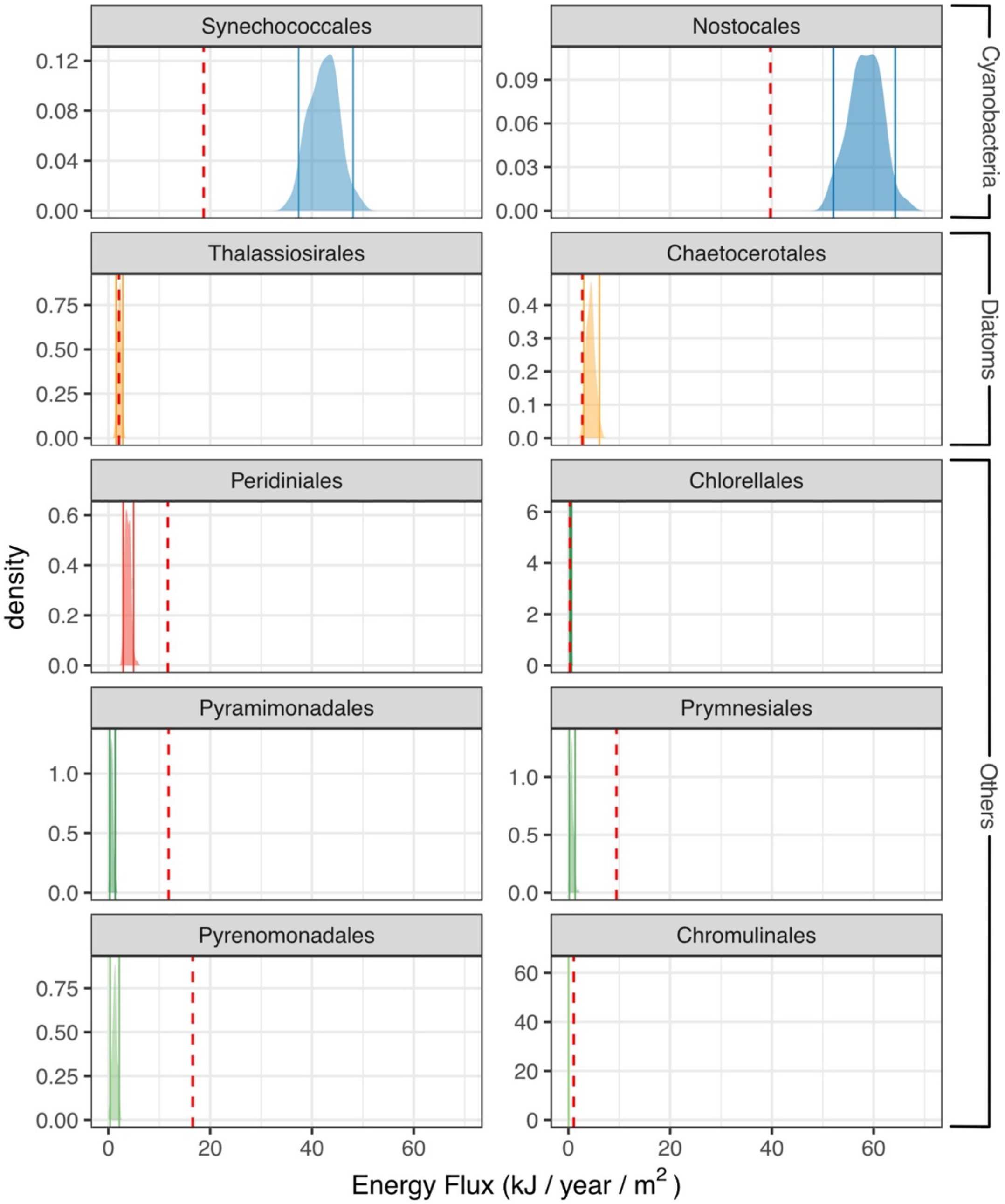

Fig. S2: The influence of uncertainty in prey selectivity on modeled annual secondary production.

To evaluate the influence of uncertainty in prey selectivity on the food web model, we calculated each primary producer's average contribution to secondary production by bootstrapping the selectivity dataset. Density plots show the posterior distribution of average detected energy fluxes over the 100 bootstrap replicates, and solid lines indicates 95% confidence interval of the mean detected energy flux (contribution to primary production). The red dashed lines indicate a null-model scenario that assumes no specific selectivity between predators and prey. The figure indicates that cyanobacteria are the main source of primary production, even when accounting for uncertainty of zooplankton selectivity. The figure also indicates that the model outcome is driven by feeding preferences.

Table S1: Average detected energy flux per prey.

Energy Flux ($kJ/m^2/year$)	*Temora*	*Centropages*	*Pseudocalanus*	*Acartia*	*Evadne*	*Bosmina*	*Synchaeta*	*Keratella*	**Total**
Nostocales	10.10	4.18	6.67	17.50	0.58	11.12	2.16	0.21	52.52
Peridiniales	4.23	1.12	1.95	1.93	0.00	0.09	2.49	0.00	11.81
Chaetocerotales	1.65	0.91	1.44	2.24	0.08	0.12	0.15	0.00	6.59
Thalassiosirales	1.87	1.35	0.53	1.52	0.01	0.00	0.00	0.00	5.29
Chromulinales	0.11	0.01	0.03	0.02	0.00	0.01	0.00	0.00	0.18
Pyrenomonadales	0.86	1.09	0.00	0.02	0.00	0.15	0.19	0.00	2.32
Prymnesiales	0.00	0.00	0.32	0.07	0.04	0.25	0.37	0.00	1.04
Pyramimonadales	2.63	0.09	0.03	0.40	0.00	0.04	0.00	0.00	3.19
Chlorellales	0.23	0.15	0.03	0.01	0.00	0.01	0.00	0.00	0.43
Synechococcales	20.04	5.76	0.95	2.53	0.12	1.12	0.19	0.01	30.73
**Total**	41.73	14.66	11.96	26.24	0.83	12.90	5.56	0.22	114.10

Table S2: Stations, dates, and zooplankton species used to calculate the diet preferences (selectivity indices) used in the network model. Prey composition in the water was identified at all sampling events. Each replicate indicates a barcoding library based on 5-12 pooled zooplankton individuals.

Station	Date	Species Sampled	n.	Ref.
**By31**	2017-06-19	*Acartia* spp.	2	*18 and 20*
Landsort Deep		*Centropages hamatus*	4	
Lat:58.59367		*Evadne nordmanni*	5	
Lon:18.23640		*Pseudocalanus* spp.	5	
		Temora longicornis	6	
		Synchaeta baltica	9	
	2017-08-15	*Acartia* spp.	8	
		Bosmina spp.	5	
		Centropages hamatus.	5	
		Pseudocalanus spp.	5	
		Temora longicornis	5	
		Sycnhaeta baltica	4	
		Keratella spp.	5	
	2018-03-19	*Acartia* spp.	4	
		Pseudocalanus spp.	6	
		Temora longicornis	4	
		Synchaeta baltica	5	
**By15**	2019-06-01	*Acartia* spp.	1	*This Study*
Gotland Deep		*Centropages hamatus*	4	
Lat:57.31222		*Temora longicornis*	2	
Lon:20.07620		*Evadne nordmanni*	1	
	2020-09-10	*Acartia* spp.	3	
		Centropages hamatus	3	
		Temora longicornis	1	
**By5**	2019-06-15	*Acartia* spp.	3	
Bornholm Deep		*Centropages hamatus*	4	
Lat:55.24998		*Temora longicornis*	5	
Lon:15.98420		*Evadne nordmanni*	3	
	2020-09-09	*Acartia* spp.	2	
		Centropages hamatus	4	
		Temora longicornis	2	

Table S3: Primers and sequences used in this study.

Name	Sequence	Function	Reference
341F	[Adapter1]-CCTACGGGNGGCWGCAG	*16S V3-V4* Forward primer	43-44
805R	[Adapter2]-GACTACHVGGGTATCTAATCC	*16S V3-V4* Reverse primer	43-44
Adapter1	ACACTCTTTCCCTACACGACGCTCTTCCGATCT	Linking outer and inner PCR	44
Adapter2	AGACGTGTGCTCTTCCGATCT	Linking outer and inner PCR	44
Handle1	AATGATACGGCGACCACCGAGATCTACAC-[IndexF]-[Adapter1]	Forward read Illumina handle	44
Handle2	CAAGCAGAAGACGGCATACGAGAT-[IndexR]-[Adapter2]	Reverse read Illumina handle	44
Index	NNNNNN<br>16 unique forward sequences and 15 unique reverse sequences	Unique hexamer combination for sample identification.	44

Data S1: All data, code, and a tutorial needed to build the model and reproduce all figures and results from this study are deposited in the Zenodo repository available at https://doi.org/10.5281/zenodo.7586799.
